# Supplementary material for: Comparison of Simple RNA Extraction Methods for Molecular Diagnosis of Hepatitis C Virus in Plasma
Source: Diagnostics (Basel). 2022 Jun 30;12(7):1599. doi: 10.3390/diagnostics12071599 (PMC9322174; doi:10.3390/diagnostics12071599)
Supplement: Supplementary file 1 [file diagnostics-12-01599-s001.zip › diagnostics-1777352-supplementary.pdf]

Table S1. Clinical efficacy evaluation of the viral RNA extraction methods for HCV detection using real time RT-PCR and RT-LAMP by samples.

No.	HCV viral load (IU/mL)	HCV genotype	Extraction methods					
			Silica-membrane based		Magnetic beads-based		Boiling with DEPC-treated distilled water	
			Real time RT-PCR, Ct	RT-LAMP	Real time RT-PCR, Ct	RT-LAMP	Real time RT-PCR, Ct	RT-LAMP
1	3,790,430.50	6(C-I)	23.16	+	20.58	+	27.17	+
2	2,257,747.6	1a	27.10	+	24.03	+	30.05	-
3	1,787,583.1	6n	23.81	+	22.55	+	28.22	+
4	1,702,037.2	3b	22.94	+	22.68	+	29.12	-
5	1,511,291.6	6(C-I)	24.30	+	22.34	+	28.34	-
6	1,374,162	3a	23.66	+	24.13	+	30.73	-
7	1,261,458	6(C-I)	27.36	+	23.87	+	29.60	+
8	1,201,254.4	3a	24.88	+	23.24	+	30.03	-
9	1,152,457.2	1a	26.52	+	25.66	+	30.80	-
10	1,003,354.7	3a	25.74	+	22.83	+	30.98	-
11	988,378.8	1a	25.36	+	24.41	+	31.17	+
12	727,803.5	6(C-I)	26.31	+	23.17	-	30.27	-
13	725,685.2	6(C-I)	24.44	+	22.90	+	28.80	+
14	709,781.9	1a	26.51	+	24.11	+	30.82	-
15	596,314.5	1a	26.35	+	24.62	+	30.07	+
16	507,432	6(C-I)	27.74	+	24.81	+	30.51	-
17	377,989.7	1a	28.92	+	25.65	+	30.15	-
18	301,989.8	1b	27.04	+	24.11	+	31.05	-
19	237,259.8	6(C-I)	28.08	+	24.79	+	31.39	-
20	170,055.7	3b	27.17	-	24.62	+	31.71	-
21	164,999.6	1	26.76	+	24.41	-	32.65	-
22	154,948.8	3a	25.93	+	24.69	+	31.49	-
23	144,706.1	1b	27.36	+	25.99	+	30.36	+
24	127,786.5	3a	26.72	+	25.99	+	31.53	-
25	80,145.6	3a	28.07	+	26.42	+	33.90	-
26	77,723	3b	29.99	+	26.39	+	31.80	-
27	54,251.4	1b	28.70	+	27.47	+	32.60	-
28	53,385	1a	30.61	+	28.76	+	Undetectable	-
29	33,765.1	1b	29.86	+	28.89	-	34.40	-
30	31,796	3a	27.71	-	26.61	-	34.81	-
31	27,369.1	6	31.83	-	29.09	-	Undetectable	-
32	20,572	1b	30.64	+	27.53	+	33.30	-
33	20,083.6	1a	32.86	+	29.62	-	32.61	-
34	18,792.4	3b	29.63	+	27.64	+	34.25	-
35	18,195	1a	30.26	-	28.68	+	35.44	-
36	17,142.1	3	29.84	-	28.39	+	34.73	-
37	16,293.2	3a	29.44	+	27.31	-	33.68	-
38	13,800.1	3a	28.95	-	27.69	+	34.46	-
39	8,443.1	3	32.55	-	30.55	-	Undetectable	-
40	7,911.2	1b	30.71	-	31.14	-	Undetectable	-
41	7,378.8	3b	31.62	-	30.34	-	Undetectable	-
42	6,196.6	6(C-I)	35.29	-	31.43	-	Undetectable	-
43	6,018.5	1	33.63	-	31.87	-	Undetectable	-
44	5,070.4	3a	30.87	-	29.58	-	Undetectable	-
45	4,559.9	1a	31.57	-	33.18	-	33.60	-
46	4,191.9	3	31.65	+	29.47	-	34.62	-
47	2,669.8	3a	34.36	-	31.67	-	Undetectable	-
48	2,140.2	1a	33.99	-	33.28	-	Undetectable	-
49	1,828	3a	32.35	-	30.40	-	Undetectable	-
50	1,764.9	3a	32.64	-	31.63	-	Undetectable	-
**Total detected (N=50)**			**50**	**33**	**50**	**31**	**38**	**7**

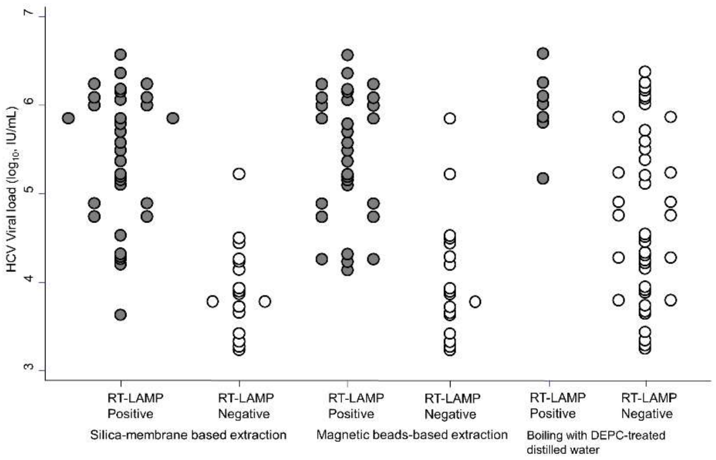

Figure S1. RT-LAMP results of all 50 clinical samples tested, according to the RNA extraction methods.
